# Innate Immune Activation Can Trigger Experimental Spondyloarthritis in HLA-B27/Huβ2m Transgenic Rats

**DOI:** 10.3389/fimmu.2017.00920

**Published:** 2017-08-07

**Authors:** Melissa N. van Tok, Nimman Satumtira, Martha Dorris, Desirée Pots, Gleb Slobodin, Marleen G. van de Sande, Joel D. Taurog, Dominique L. Baeten, Leonie M. van Duivenvoorde

**Affiliations:** ^1^Clinical Immunology and Rheumatology, Amsterdam Rheumatology and Immunology Center, Academic Medical Center, University of Amsterdam, Amsterdam, Netherlands; ^2^Experimental Immunology, Academic Medical Center, University of Amsterdam, Amsterdam, Netherlands; ^3^Rheumatic Diseases Division, Department of Internal Medicine, University of Texas Southwestern Medical Center, Dallas, TX, United States; ^4^Internal Medicine, Bnai Zion Medical Center, Haifa, Israel

**Keywords:** spondyloarthritis, innate immunity, HLA-B27 transgenic rats, inflammation, bone formation

## Abstract

Spondyloarthritis (SpA) does not display the typical features of auto-immune disease. Despite the strong association with MHC class I, CD8^+^ T cells are not required for disease induction in the HLA-B27/Huβ2m transgenic rats. We used Lewis HLA-B27/Huβ2m transgenic rats [21-3 × 283-2]F1, HLA-B7/Huβ2m transgenic rats [120-4 × 283-2]F1, and wild-type rats to test our hypothesis that SpA may be primarily driven by the innate immune response. *In vitro*, splenocytes were stimulated with heat-inactivated *Mycobacterium tuberculosis* and cytokine expression and production was measured. *In vivo*, male and female rats were immunized with 30, 60, or 90 µg of heat-inactivated *M. tuberculosis* and clinically monitored for spondylitis and arthritis development. After validation of the model, we tested whether prophylactic and therapeutic TNF targeting affected spondylitis and arthritis. *In vitro* stimulation with heat-inactivated *M. tuberculosis* strongly induced gene expression of pro-inflammatory cytokines such as TNF, IL-6, IL-1α, and IL-1β, in the HLA-B27 transgenic rats compared with controls. *In vivo* immunization induced an increased spondylitis and arthritis incidence and an accelerated and synchronized onset of spondylitis and arthritis in HLA-B27 transgenic males and females. Moreover, immunization overcame the protective effect of orchiectomy. Prophylactic TNF targeting resulted in delayed spondylitis and arthritis development and reduced arthritis severity, whereas therapeutic TNF blockade did not affect spondylitis and arthritis severity. Collectively, these data indicate that innate immune activation plays a role in the initiation of HLA-B27-associated disease and allowed to establish a useful *in vivo* model to study the cellular and molecular mechanisms of disease initiation and progression.

## Introduction

Spondyloarthritis (SpA) is the second most frequent form of chronic inflammatory arthritis and lacks many of the prototypical features of auto-immune diseases. Due to the strong association with HLA-B27, classification as “mixed disease” in the spectrum ranging from auto-immune to auto-inflammatory was suggested (1). Based on a series of translational studies, we more recently proposed that auto-immune mechanisms (relating to auto-reactive T and/or B cells) are unlikely to play a role in the pathogenesis of SpA. Therefore, we suggest that hyper-reactive innate immune responses can drive SpA (2, 3). This hypothesis, however, needs to be reconciled with the central role of HLA-B27 in SpA pathogenesis. Besides the traditional antigen-presenting role of HLA-B27 activating CD8^+^ T cells (4), two alternative hypotheses emerged that proposed innate, antigen-independent mechanisms by which HLA-B27 could trigger inflammation in SpA (5). Allen et al. proposed that HLA-B27 β2m-free heavy chain homodimers formed on the cell surface, can bind to specific KIR-receptors on NK cells and T cells and thereby trigger the production of pro-inflammatory cytokines, including IL-17 (6–10). Alternatively, Mear et al. proposed that HLA-B27 has a particular propensity to misfold in the ER (11), and initiate an unfolded protein response resulting in the production of pro-inflammatory cytokines such as IL-23 (12). Interestingly, recent clinical trials have demonstrated that, besides TNF, the IL-23/IL-17 axis plays a central role in human SpA (13–15). The non-mutually exclusive pathways that may contribute to HLA-B27-associated disease, remain difficult to delineate in human patients (16, 17).

The HLA-B27/Huβ2m transgenic rat model provides a powerful tool to assess the relative contribution of acquired and innate immune responses in SpA. In the original HLA-B27/Huβ2m transgenic rat model first described in 1990, overexpression of HLA-B27 and Huβ2m led to spontaneous multi-system inflammatory disease (18). The second model with less HLA-B27 copy numbers but additional copy numbers of Huβ2m was first described in 2006. These rats, in the absence of colitis or other systemic inflammation, developed spontaneous spondylitis and arthritis at the age of 9 months, with an increased, but still low, incidence (40% of the male rats while females remain healthy) (19, 20). Studies assessing the potential role of HLA-B27 in the activation of pathogenic T cells demonstrated that CD4^+^ T cells from the original HLA-B27 transgenic rats efficiently induced SpA symptoms (21), while CD8^+^ T cells were not required for disease in both the original and the second HLA-B27 transgenic rat model (22, 23). In contrast, rederiving the original HLA-B27 transgenic rats in a germ-free environment prevented the inflammatory phenotype (24), which fits with the human observation that gastrointestinal infections with, amongst others, Salmonella, Shigella, and Campylobacter, can trigger SpA in HLA-B27-positive individuals (25). Furthermore, in the second HLA-B27 transgenic rat model epididymo-orchitis is preceding the development of spondylitis and arthritis in 100% of the rats (19), while orchiectomy completely prevents spontaneous spondylitis and arthritis development in these rats (20). These data suggest that danger signals such as GI pathogens and testicular tissue inflammation trigger disease in HLA-B27 transgenic rats, we hypothesized that the presence of HLA-B27 would increase the sensitivity toward innate immune activation and lower the threshold for spondylitis and arthritis development. To test this hypothesis, we used the second HLA-B27/Huβ2m transgenic rat model (further referred to as HLA-B27 tg rats) and the HLA-B7/Huβ2m transgenic control rats (further referred to as HLA-B7 tg rats).

## Materials and Methods

### *In Vitro* Stimulation Assays

Spleens from HLA-B27 tg, HLA-B7 tg, or wild-type rats (*n* = 6 rats/group) were collected and mononuclear cells were isolated using Ficoll. Cells were stimulated with 1, 5, or 25 µg/ml heat-inactivated *Mycobacterium tuberculosis*, 5 µg/ml zymosan, or 50 ng/ml LPS, using unstimulated samples as controls. Gene expression was measured in duplex reactions using SYBR green primers (sequences are available upon request). The relative expression was calculated with the “2^−ddCt^ method” (26). For protein analysis, supernatant was collected and analyzed by ELISA for TNF, IL-6, IL-1β (R&D) according to the manufacturer’s protocol.

### Rats

The transgenic 21-3 (HLA-B27/Huβ2m), 120-4 (HLA-B7/Huβ2m), and 283-2 (Huβ2m) rat lines, all on the inbred Lewis background, were maintained in our breeding facility at UTSWMC (Dallas, TX, USA) or AMC (Amsterdam, The Netherlands). For experiments, F1 rats [21-3 × 283-2] and/or [120-4 × 183-2], both males and females, were used at 6–12 weeks of age. Rats were housed, three to four per cage, either conventionally or in individually ventilated cages with appropriate cage enrichment and *ad libitum* access to water and chow. All animal experiments were performed either at UTSWMC or at AMC and were approved by the Institutional Animal Care and Use Committee.

### Immunization

6-week-old male and female rats were immunized with 30–90 µg of pulverized heat-inactivated *M. tuberculosis* (Difco, Detroit, MI, USA) in 100 µl incomplete Freund’s adjuvant (Chondrex) *via* subcutaneous injection in the tail base.

### Clinical Scoring

The presence of arthritis in the paws was determined macroscopically and digital swelling was measured with plethysmometry where stated. Arthritis severity in each paw was graded 0–3: 0 = normal joints, 1 = 1 swollen joint, 2 = 2 or more swollen joints, and 3 = extreme swelling of the entire paw and/or ankylosis. Cumulative clinical scores per rat were calculated and used for severity analysis. Swelling (cubic centimeter) was normalized either to the days before disease onset in case of prophylactic treatment or to the day of start treatment in case of therapeutic treatment. Spondylitis was determined macroscopically (yes/no). In case of severe disease, rats were sacrificed due to ethical considerations, with the last observation carried forward. Severe disease was defined by 15% bodyweight loss or two completely swollen paws for validation experiments or prophylactic treatment; and 20% bodyweight loss or four completely swollen paws for therapeutic treatment. During treatment experiments, scoring was performed by a blinded observer.

### Orchiectomy

Surgery was performed using standard methods (Protocol Envigo, Horst, The Netherlands). In brief, 4-week-old rats received 5 mg/kg rimadyl 15 min prior to surgery, rats were anesthetized with isoflurane + 1 l/min O_2_. A medial skin incision of 2–3 cm was made, caudal to rostral and a similar size incision was made in the peritoneum, over the Linea Alba. Both testicles, including epididymis and VAS were removed and the peritoneum and skin were sutured.

### Treatment with Thalidomide or Etanercept

Rats received 150 mg/kg thalidomide or PBS daily *via* oral gavage (*n* = 9/group) in a prophylactic experiment. In separate experiments, rats received 10 mg/kg etanercept or PBS twice weekly *via* subcutaneous injection, prophylactically (*n* = 6/group) or therapeutically (*n* = 5–6/group). Prophylactic treatment started 7 days after immunization, therapeutic treatment started 1 week after 50% arthritis incidence. Treatment continued for 5 weeks in all experiments. In case of therapeutic treatment, rats were randomized based on arthritis score on the first day of treatment.

### Histological Analysis

Paws and tail tissues were fixed, and sectioned as previously described (27). Sections were stained for hematoxylin and eosin or safranin O/fast green. Stained sections were semi-quantitatively scored by two independent, blinded observers (Melissa N. van Tok and Leonie M. van Duivenvoorde). Sections were scored 0–3 for inflammation, destruction, new bone formation, and hypertrophic chondrocytes (0 = normal, 1 = mildly affected, 2 = moderately affected, and 3 = severely affected). In the validation studies, histological scores from HLA-B27 transgenic males immunized with 30 µg heat-inactivated *M. tuberculosis* were pooled together with the females immunized with 60 µg heat-inactivated *M. tuberculosis* (*n* = 10). From the HLA-B7 transgenic rats, the rats immunized with matching doses (30 µg for males, 60 µg for females) of heat-inactivated *M. tuberculosis* were used to compare with (*n* = 6). In the therapeutic treatment experiment with etanercept, tissue from age-matched healthy controls was taken along.

### Statistical Analysis

Data were statistically analyzed using GraphPad Prism software. Values of *p* < 0.05 were considered significant. For comparison between two groups, a Mann–Whitney *U*-test was performed, for multiple group comparison we used a two-way ANOVA or a Kruskal–Wallis test and for the survival graphs (incidence) we used a log-rank test.

## Results

### Hyper-Responsiveness of HLA-B27-Expressing Cells upon Innate Immune Activation

**Figure 1:**
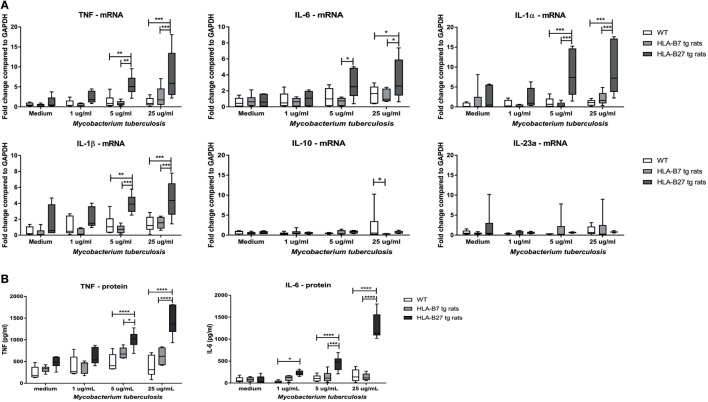
Increased pro-inflammatory cytokine expression and secretion after innate immune activation in HLA-B27 tg rats. **(A)** mRNA expression of cytokines was analyzed after *in vitro* stimulation with heat-inactivated *M. tuberculosis* in HLA-B27 tg rats and controls. **(B)** TNF and IL-6 production were confirmed on protein level by ELISA. Data are min–max. *p < 0.05, **p < 0.01, ***p < 0.001, ****p < 0.0001.

To test the hypothesis that HLA-B27 overexpression increases the sensitivity toward innate immune activation, splenocytes of non-immunized, non-diseased HLA-B27 tg rats, HLA-B7 tg rats, and wild-type control rats (*n* = 6/group) were stimulated with 1, 5, or 25 µg/ml heat-inactivated *M. tuberculosis* (Figure 1A). Unstimulated splenocytes had low but similar mRNA expression of all measured cytokines in all three rat genotypes. Splenocytes of HLA-B27 tg rats showed significantly increased *tnf, il1a, il1b*, and *il6* expression upon 5 and 25 µg/ml *M. tuberculosis* stimulation compared with both controls. *Il23a* (p19) and *il10* expression were not increased. These data were confirmed at the protein level by TNF and IL-6 measurements in the supernatant (Figure 1B), with IL-1β being low to undetectable in all conditions (data not shown). As *M. tuberculosis* can activate a variety of innate immune receptors (28) as well as antigen-specific adaptive immune responses (29), we confirmed these observations by showing a similar increase in pro-inflammatory cytokine production upon stimulation of HLA-B27 tg rat-derived versus control splenocytes with the TLR2/dectin-1 ligand zymosan, but not with the TLR4 ligand LPS (Figure S1 in Supplementary Material). Taken together, *in vitro* innate immune activation—in particular through TLR2/dectin-1—causes a significant upregulation of pro-inflammatory cytokines in the HLA-B27 tg rat splenocytes.

### *In Vivo* Immunization with Heat-Inactivated *M. tuberculosis* Triggers Spondylitis and Arthritis in HLA-B27 tg Rats

Male HLA-B27 tg rats spontaneously develop spondylitis and arthritis with an incidence of 40 and 70%, respectively, and a variable disease onset between 4 and 9 months of age. Female rats on the other hand are not capable of spontaneous spondylitis and arthritis development (19). Based on the upregulation of pro-inflammatory cytokine production *in vitro*, we hypothesized that *in vivo* activation of the innate immune system would trigger spondylitis and arthritis development in the HLA-B27 tg rats. Heat-inactivated *M. tuberculosis* was selected for immunization, based on the arthritis model adjuvant induced arthritis (AIA). It is commonly known that wild-type LEWIS rats do not develop spondylitis after immunization with (low doses of) heat-inactivated *M. tuberculosis*. Due to ethical considerations, no wild types were included in the *in vivo* experiments. HLA-B27 and HLA-B7 tg males and females were immunized with low doses of heat-inactivated *M. tuberculosis* and monitored for clinical spondylitis and arthritis development (Table 1). In male rats, immunization with higher doses of heat-inactivated *M. tuberculosis* (60 and 90 µg) induced spondylitis and arthritis in both HLA-B27 tg rats and HLA-B7 tg controls. Upon immunization with 30 µg of heat-inactivated *M. tuberculosis*, however, none of the HLA-B7 tg rats developed disease whereas 100 and 80% of the HLA-B27 tg males developed spondylitis and arthritis, respectively. To confirm and extend these findings, we first performed similar experiments in female HLA-B27 tg rats which, in contrast to male rats, do not develop spontaneous spondylitis and arthritis (19, 20). None of the HLA-B7 tg female control rats developed spondylitis or arthritis upon immunization with heat-inactivated *M. tuberculosis* up to a dose of 90 µg. In contrast, all female HLA-B27 tg rats immunized with 60 or 90 µg heat-inactivated *M. tuberculosis* developed both spondylitis and arthritis. Epididymo-orchitis has been shown to be a pre-requisite for the development of spontaneous spondylitis and arthritis in the HLA-B27 tg rat (20). We confirmed that innate immune activation triggers spondylitis and arthritis by demonstrating that immunization with 30 or 60 µg of heat-inactivated *M. tuberculosis* could overcome the protective effect of orchiectomy in male rats. Immunization with 30 or 60 µg heat-inactivated *M. tuberculosis* was sufficient to induce both spondylitis and arthritis in all HLA-B27 transgenic males. Collectively, these data indicate that innate immune activation using heat-inactivated *M. tuberculosis* immunization triggers spondylitis and arthritis development in HLA-B27 transgenic rats, including disease-resistant female and orchiectomized male rats.

**Table 1:** Spondylitis and arthritis incidence in immunized rats.

Sex	Tg	*Mycobacterium tuberculosis* (μg)	Spondylitis		Arthritis	
			*n* (%)	Mean onset (days)	*n* (%)	Mean onset (days)
Males	HLA-B7	30	0/3 (0)	–	0/3 (0)	–
		60	2/3 (67)	23	2/3 (67)	20
		90	3/3 (100)	19	2/3 (67)	14
	HLA-B27	30	7/7 (100)	23	6/7 (80)	22
		60	2/2 (100)	23	2/2 (100)	20
		90	2/2 (100)	21	2/2 (100)	22
Females	HLA-B7	30	0/3 (0)	–	0/3 (0)	–
		60	0/3 (0)	–	0/3 (0)	–
		90	0/3 (0)	–	0/3 (0)	–
	HLA-B27	30	1/3 (33)	38	1/3 (33)	26
		60	3/3 (100)	16	3/3 (100)	15
		90	3/3 (100)	14	3/3 (100)	15
Orchiectomized males	HLA-B27	30	3/3 (100)	28	3/3 (100)	24
		60	3/3 (100)	23	3/3 (100)	21

### Validation of the Innate Immune-Induced Experimental SpA Model in HLA-B27 tg Rats

**Figure 2:**
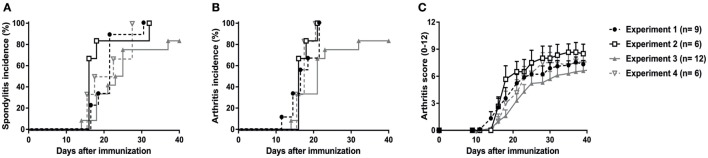
Validation and reproducibility of *M. tuberculosis*-induced spondylitis and arthritis in HLA-B27 tg rats. Male and female rats were immunized with 30–60 µg *M. tuberculosis* in four independent experiments. Incidence of **(A)** spondylitis and **(B)** arthritis (data are % of diseased) as well as **(C)** arthritis scores (data are mean ± SEM) were analyzed and showed to be comparable in all experiments.

The previous *in vivo* pilot experiments were performed in the animal facility of UTSW Medical Center in Dallas, TX, USA (except for the orchiectomy experiment). To confirm our findings and validate the reproducibility and robustness of the model, the experiments were repeated, independently in the animal facility of the AMC in Amsterdam, The Netherlands. A total of 33 HLA-B27 tg rats were immunized with heat-inactivated *M. tuberculosis* (30 µg in 24 males and 60 µg in 9 females) in four independent experiments. Due to ethical considerations, wild types or non-immunized HLA-B27 transgenic rats were not included in these experiments as it has repeatedly been shown that low amounts of heat-inactivated *M. tuberculosis* (30–60 µg) did not induce spondylitis in wild types (30–32). Non-immunized females or orchiectomized males do not develop disease (19, 20). In all four experiments, 80–100% of the rats developed spondylitis (Figure 2A) and arthritis (Figure 2B) with a disease onset between days 14 and 21 after immunization (Figure 2C). Clinical scoring of the arthritis (0–3 per paw with a maximum score of 12 per rat), revealed a similar arthritis severity in all four experiments with an average score of 8 (Figure 2C). These results confirm that immunization with heat-inactivated *M. tuberculosis* reproducibly and predictably triggers clinical spondylitis and arthritis in HLA-B27 tg rats.

### Innate Immune Activation Triggers Not Only Inflammation But Also Joint Destruction and New Bone Formation As Histologically Detected in HLA-B27 tg Rats

**Figure 3:**
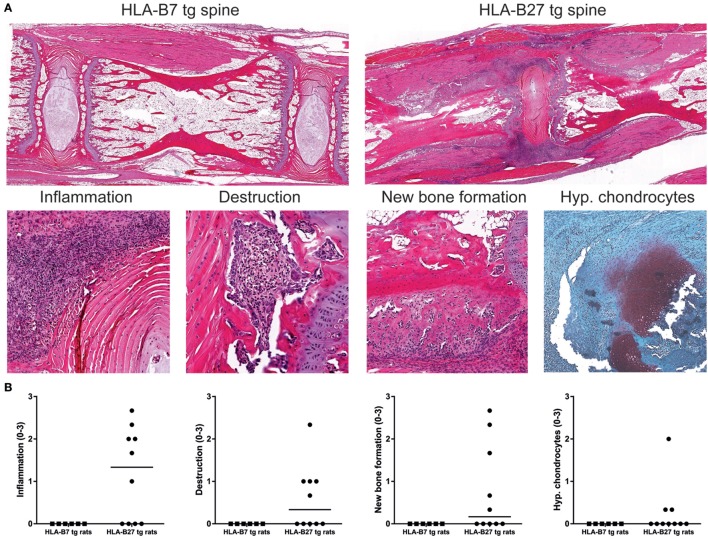

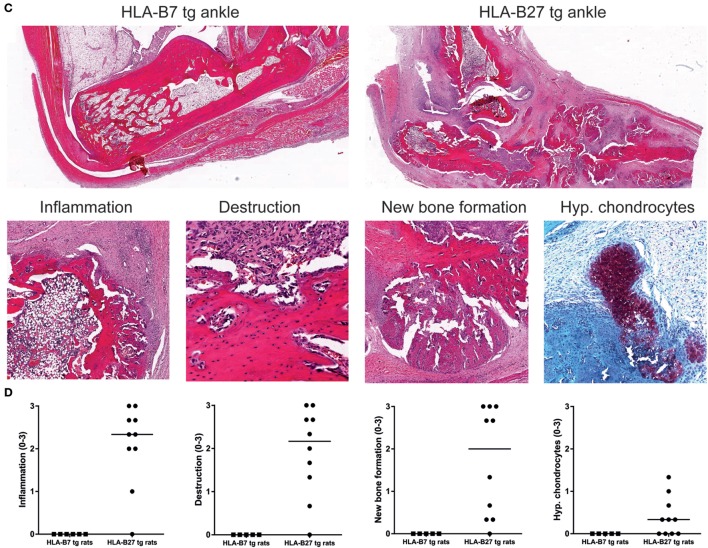
Histopathology in HLA-B27 tg rats immunized with *M. tuberculosis* was comparable with the spontaneous disease model. Spine and ankle joints were histologically analyzed. **(A)** In an overview of HLA-B7 and HLA-B27 tg rats (20× magnification), spinal inflammation (80×), destruction (200×), periosteal new bone formation (80×), and hypertrophic chondrocytes (80×) was only detected in HLA-B27 tg rats, **(B)** as quantified (pooled males and females; data are medians). **(C)** An overview of ankle joints (20×) showed inflammation (40×), destruction (200×), new bone formation (40×), and hypertrophic chondrocytes (80×) again only in HLA-B27 tg rats, **(D)** as quantified (pooled males and females; data are medians).

HLA-B27 tg rats that spontaneously develop spondylitis and arthritis show histopathological features of inflammation, cartilage and bone destruction, and in particular new bone formation in axial and peripheral joints (27). Histological analysis of spine and ankles was done to evaluate whether similar histopathology is present upon immunization with heat-inactivated *M. tuberculosis*. In the spine, we detected inflammatory cell infiltration around the intervertebral discs, vertebral endplate destruction, periosteal new bone formation, and ectopic foci of hypertrophic chondrocytes in HLA-B27 tg rats but not in HLA-B7 tg controls (Figures 3A,B). Similarly, the ankle joints were histologically normal in HLA-B7 tg rats but depicted marked articular and peri-articular inflammation, cortical bone destruction, periosteal new bone formation, and hypertrophic chondrocytes in HLA-B27 tg (Figures 3C,D). The axial and peripheral histopathology was similar to what we previously described in a cross sectional study of spontaneous spondylitis and arthritis in male HLA-B27 tg rats including, unlike what is seen in classical arthritis models, typical spinal involvement, and new bone formation (27).

### Thalidomide Treatment Partially Protects from Experimental SpA in HLA-B27 tg Rats

**Figure 4:**
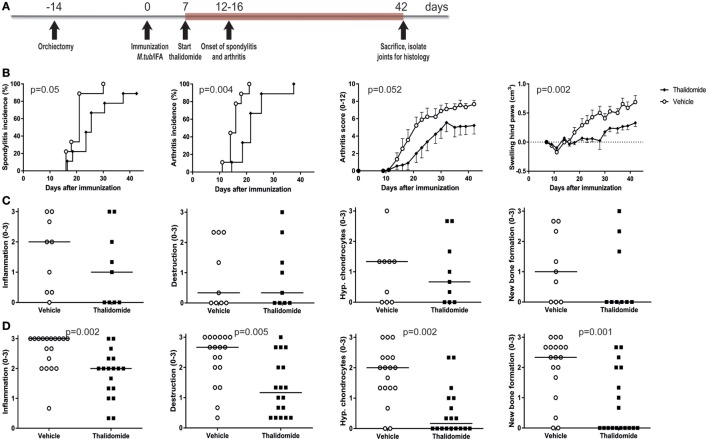
Prophylactic treatment with thalidomide delayed spondylitis and arthritis development and reduced arthritis severity. **(A)** HLA-B27 tg rats were prophylactically treated with thalidomide. Treatment started 7 days after immunization and continued for 5 weeks. **(B)** Clinical results include spondylitis and arthritis incidence, arthritis severity, and hind paw swelling. Data are mean ± SEM. Histologically inflammation, destruction, periosteal new bone formation, and hypertrophic chondrocytes in **(C)** spine and **(D)** ankle joints were quantified. Data are medians.

The high disease incidence of 80–100% in both male and female rats, the predictable onset between days 14 and 21, and the robust clinical and histopathological features provided the opportunity to perform intervention studies in the heat-inactivated *M. tuberculosis*-induced disease in HLA-B27 tg rats. To validate this concept, rats were prophylactically treated with thalidomide, a small molecule targeting, amongst others, the TNF pathway (33). Treatment with thalidomide or vehicle started 1 week after immunization and continued daily for 5 weeks (Figure 4A). Spondylitis and arthritis development were significantly delayed and arthritis severity had the tendency toward a decrease in the thalidomide treated rats when compared with the vehicle treated rats (Figure 4B). Histopathological analysis of the four disease features (inflammation, destruction, new bone formation, and hypertrophic chondrocytes) confirmed a trend toward lower scores in the spine (Figure 4C) and significantly scores in the ankle joints (Figure 4D) in thalidomide versus vehicle treated rats. These data demonstrated that the model is suitable for intervention studies, allowing us to use targeted therapies to establish the role of key innate pathways in the HLA-B27 associated disease process.

### Prophylactic, But Not Therapeutic, Targeting of the TNF Pathway with Etanercept Suppresses Experimental SpA in HLA-B27 tg Rats

**Figure 5:**
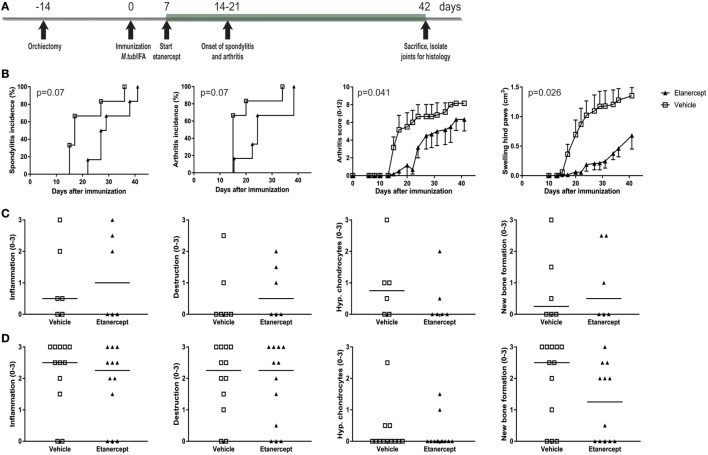
Prophylactic treatment with etanercept confirmed clinically delayed spondylitis and arthritis development and reduced arthritis severity. **(A)** HLA-B27 tg rats were prophylactically treated with etanercept. Treatment started 7 days after immunization and continued for 5 weeks. **(B)** Clinical results include spondylitis and arthritis incidence, arthritis severity, and hind paw swelling. Data are mean ± SEM. Histologically inflammation, destruction, new bone formation, and hypertrophic chondrocytes in **(C)** spine and **(D)** ankle joints were quantified. Data are medians.

TNF is one of the major pro-inflammatory cytokines in innate immune inflammation and plays a crucial role in human SpA. We therefore tested whether TNF inhibition with etanercept could prevent and/or treat experimental SpA in the heat-inactivated *M. tuberculosis*-induced disease in HLA-B27 tg rats. Similarly to thalidomide, etanercept treatment was started 1 week after immunization but before onset of clinical disease (Figure 5A). Etanercept treatment delayed the appearance of spondylitis and arthritis and suppressed arthritis severity (Figure 5B). Inflammation, destruction, new bone formation, and hypertrophic chondrocytes were not different between the two groups, both in the spine (Figure 5C) and in the ankle joints (Figure 5D).

**Figure 6:**
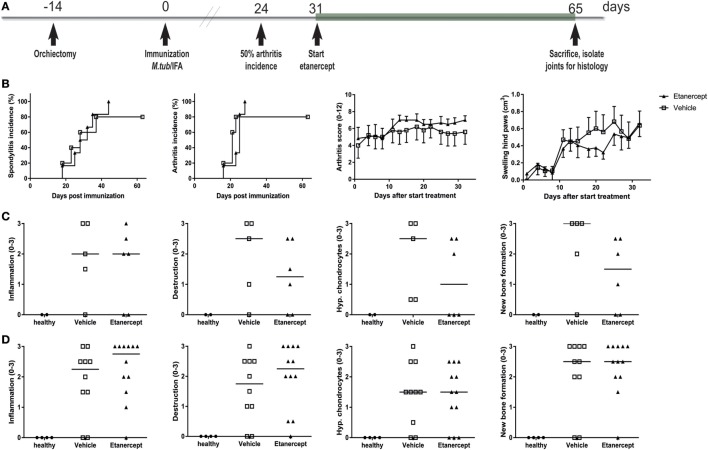
Therapeutic treatment with etanercept did not impact disease incidence and severity. **(A)** HLA-B27 tg rats were therapeutically treated with etanercept. Treatment started 7 days after 50% arthritis incidence and continued for 5 weeks. **(B)** Clinical results include spondylitis and arthritis incidence, arthritis severity, and hind paw swelling. Data are mean ± SEM. Histologically inflammation, destruction, new bone formation, and hypertrophic chondrocytes in **(C)** spine and **(D)** ankle joints were quantified. Data are medians.

In contrast to prophylactic treatment, therapeutic treatment with etanercept, initiated at day 31 (Figure 6A), did not affect incidence or severity of clinical disease (Figure 6B). The absence of therapeutic effect in this setting was confirmed by histological analysis, where in contrast to the age-matched healthy controls both vehicle and etanercept treated rats presented with histopathological processes of SpA in the spine (Figure 6C) and the ankles (Figure 6D), as semi-quantitatively assessed. Collectively, these data provide evidence that experimental SpA in HLA-B27 tg rats is, at least partially, TNF dependent and suggests that the role of this key innate immune cytokine is more important in the induction phase than in established disease in this particular model.

## Discussion

In the present study, we used the HLA-B27/Huβ2m transgenic rats [21-3 × 283-2]F1 to test the hypothesis that innate immune pathways are key drivers of SpA. We showed here that innate immune activation of splenocytes increases expression and production of pro-inflammatory cytokines in HLA-B27 tg rats when compared with HLA-B7 tg controls and Lewis wild-type rats. *In vivo*, innate immune activation with low doses of heat-inactivated *M. tuberculosis* triggered spondylitis and arthritis development, increased arthritis severity, and synchronized disease onset in HLA-B27 tg males and females. Moreover, immunization could overcome the protective effect of orchiectomy in male HLA-B27 tg rats. The induced SpA-like disease in HLA-B27 tg rats was highly reproducible, showed all prototypical pathological features of inflammation, destruction, and remodeling observed in the spontaneous HLA-B27 tg rat model, and could be partially suppressed by TNF blockade during its induction phase. Collectively, these data support the hypothesis that innate immunity plays a role in HLA-B27-associated disease. Herewith, we provide a novel and relevant *in vivo* model for interrogating SpA pathogenesis by targeted interventions.

The immunization strategy was partially based on the AIA model. However, in the HLA-B27 tg rats immunization 30–60 µg *M. tuberculosis* was sufficient to induce spondylitis and arthritis in male and female rats. Without immunization spontaneous development of spondylitis and arthritis appeared in 40% of the male rats around 9 months of age (20). As previously described, orchiectomy prevents the development of clinical and histological spondylitis and arthritis symptoms (which is also evident in the age-matched healthy HLA-B27 tg controls in Figure 6). Following ethical considerations, we decided not to take along non-immunized HLA-B27 tg rats as a control. Instead HLA-B7 transgenic, a negative control HLA-B allele (not associated with SpA) control, served as a more proper control. These rats were generated in a similar way as the HLA-B27 tg rats; the same construct was used and the transgene copy number and expression are similar. Disease induction following immunization with low doses of *M. tuberculosis* are thus specific for HLA-B27 tg rats, and not just as an artifact of the HLA-B overexpression.

Our data are consistent with several lines of evidence suggesting a crucial role for innate immune pathways in experimental and human SpA. In the HLA-B27 transgenic rats, the potential relevance of danger signals for disease induction was already suggested by the findings that the microbial environment was decisive for disease development in the original HLA-B27 tg rat model (24), and that orchitis preceding spondylitis and arthritis was required in the second HLA-B27 tg rat model (20). Together this suggests that a danger signal is required for the induction of disease in the HLA-B27 tg rats. Innate immune activation was previously shown to be essential for SpA-like disease development in the SKG mouse, a model characterized by spontaneous deforming arthritis induced by a genetic mutation in T cell signal transduction (34). SKG mice failed to develop arthritis in SPF conditions despite the presence of arthritogenic T cells, however stimulation with dectin-1 receptor ligands, or C5a-dependent complement activation, restored disease with a more SpA-like, peripheral and axial, phenotype (35–37). In these mice disease, development after innate triggering was demonstrated to be IL-23 dependent (37, 38). Also in humans, the development of SpA can be triggered by danger signals, with as prototypical example reactive arthritis. In this SpA subtype, disease appears 4–6 weeks after severe gastrointestinal infections with *Campylobacter, Salmonella, Shigella*, or *Yersinia*, or urogenital infections with *Chlamydia trachomatis*. A number of other infectious agents were implicated to trigger reactive arthritis, including intestinal parasites (39). The post-infectious arthritis is more likely to evolve into chronic SpA in HLA-B27^+^ individuals (25, 40), a progression possibly related to TLR2 polymorphisms (41). Moreover, in response to LPS, macrophages from AS patients had increased IL-23 and TNF production (42).

Whereas the data of the current study globally support the concept of an interplay between innate immune activation and HLA-B27 in the pathogenesis of SpA, a number of important questions remain unanswered. First, it needs to be better defined which immune pathways play a role in the induction of HLA-B27-associated disease. *Mycobacterium tuberculosis* activates many different innate immune receptors, including TLR2, TLR4, and dectin-1 (28), and may additionally induce an adaptive immune response. We showed here that zymosan but not LPS induced a similar response as heat-inactivated *M. tuberculosis* stimulation *in vitro* (Figure S1 in Supplementary Material), leading to the hypothesis that TLR2 and/or dectin-1 may be crucial. Preliminary *in vivo* experiments in a small number of rats (Figure S2 in Supplementary Material) additionally showed that flagellin and CpG could induce experimental SpA in HLA-B27 tg animals. Altogether these data indicate that any innate trigger might induce an upregulation in pro-inflammatory cytokines and subsequent spondylitis and arthritis development. Disease induction is merely dependent on the required dose per trigger and/or the threshold within the animal. Whereas these data are all consistent with a role of innate immune activation, it is clear that further work should better delineate the mechanisms and identify the cell types involved. Our *in vitro* experiments were performed with total splenocytes and do not allow to assess the role of different innate cell populations, including macrophages, dendritic cells, NK cells, and innate immune lymphocytes. Third, the questions how HLA-B27 influences these innate immune responses, including the potential role of ER stress induction and/or KIR activation by aberrant forms of HLA-B27, and how specific this is for HLA-B27 remains to be resolved. Whereas our experiments did show a clear difference between the HLA-B27 tg rats and the control strains, it should be noted that higher doses of heat-inactivated *M. tuberculosis* could induce similar pathology in male HLA-B7 tg control rats. However, in wild-type rats it has been shown that 100–1,000 µg heat-inactivated *M. tuberculosis* is sufficient to induce only arthritis (30, 32, 43, 44). Pure adjuvants without mycobacteria could also induce arthritis in few rat strains, however not in LEWIS rats (45). Importantly, only one paper from 1961 reported the induction of spondylitis in wild-type Long–Evans rats after immunization with an enormous dose of heat-inactivated *M. tuberculosis* (five times 500 μg) (31). Due to conscious ethical considerations, wild-type rats were not included in any of the vivo experiments, since as mentioned above, low doses (30–90 µg) of heat-inactivated *M. tuberculosis* have never been reported to induce clinical spondylitis or axial pathology including new bone formation in wild-type rats (30, 32). These data thus suggest that HLA-B27 may contribute to lower the threshold for innate immune activation rather than be a required factor for SpA-like disease, which would fit with the fact that 20–30% of human SpA patients are HLA-B27-negative. Despite these caveats, one of the major outcomes of the present study is that innate immune activation made it possible to increase incidence and synchronize disease in both male and female rats. This facilitated performance of intervention studies with targeted therapies to interrogate the pathogenesis of HLA-B27-associated disease in this model. After validating this concept with thalidomide, we showed here that the model is partially TNF-dependent, in particular in the induction phase of the disease.

These data have to be replicated and confirmed, in particular using a monoclonal anti-rat TNF antibody rather than the soluble receptor construct etanercept, as contradictory data on the effects of etanercept in rats have been reported (46–49). Unfortunately, tools to block TNF in rats are limited.

Finally, this experimental SpA model opens ways for further mechanistic studies with existing and novel therapeutic compounds focused not only on inflammation but also on the relationship between inflammation and structural remodeling in SpA.

## Ethics Statement

This study was carried out in accordance with the recommendations of Institutional Animal Care and Use Committees. The protocols were approved by the Animal ethical committee of either UTSW Medical Center, Dallas, TX, USA or the Academic Medical Center, Amsterdam, The Netherlands.

## Author Contributions

MT contributed to data collection, analysis, and interpretation and was involved in writing of the manuscript. NS, MD, DP, and GS contributed to data acquisition and analysis. JT, MS, and DB contributed to study design, data interpretation and were involved in writing of the manuscript. LD contributed to study design, data collection, analysis, and interpretation and was involved in writing of the manuscript. All authors revised the manuscript and approved the submitted and published versions.

## Conflict of Interest Statement

JT has license agreements with AbbVie, Anges, Inc., and Celgene. DB is part-time employee of UCB and received consultancy fees/grants from AbbVie, Pfizer, MSD, Roche, BMS, Novartis, Eli Lilly, Janssen, Glenmark, Boehringer-Ingelheim. MT, NS, MD, DP, GS, and LD have no conflict of interest.
