# Supplementary material for: Innate Immune Activation Can Trigger Experimental Spondyloarthritis in HLA-B27/Huβ2m Transgenic Rats
Source: Front Immunol. 2017 Aug 7;8:920. doi: 10.3389/fimmu.2017.00920 (PMC5545590; doi:10.3389/fimmu.2017.00920)
Supplement: Supplementary file 1 [file Data_Sheet_1.PDF]

Supplementary

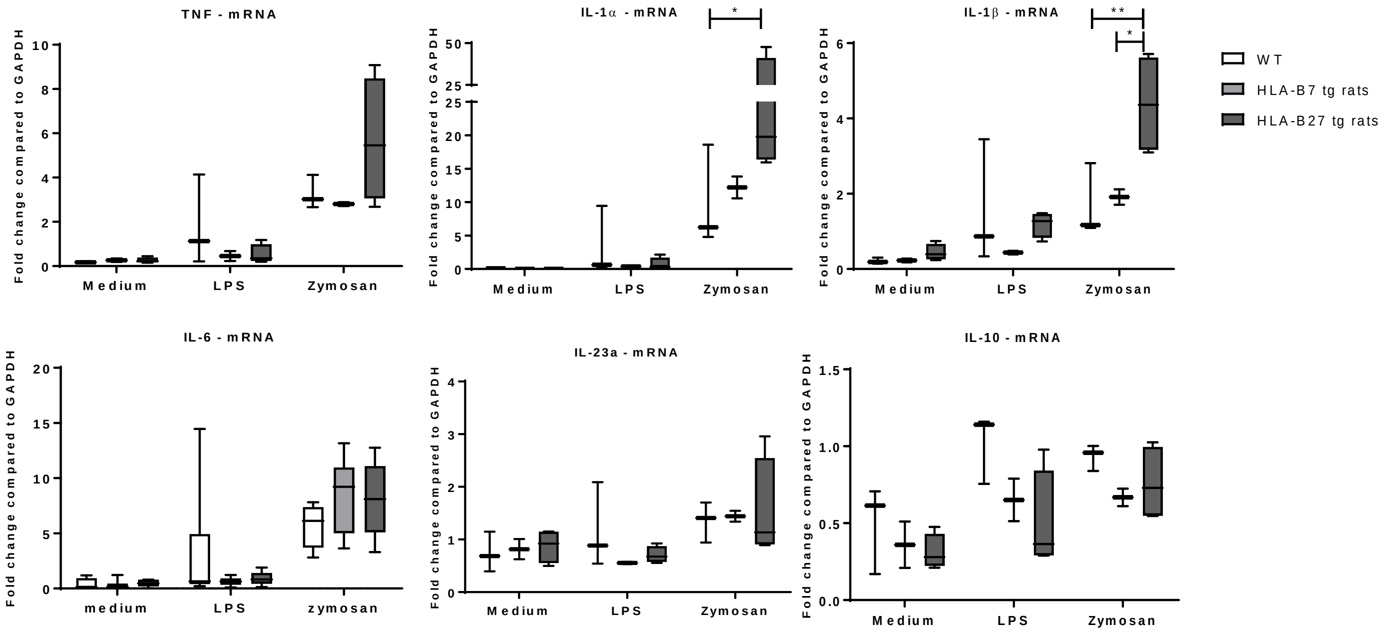

Suppl. Fig. 1 Zymosan stimulation confirmed increased expression of pro-inflammatory cytokines in HLA-B27 tg rats. Splenocytes from HLA-B27 tg rats and controls were stimulated with zymosan or LPS, gene expression for TNF, IL-1α, IL-1β, IL-6, IL-23a and IL-10 was measured by qPCR. Data are min-max. * $p<0.05$ ** $p<0.01$.

Supplementary

Flagellin

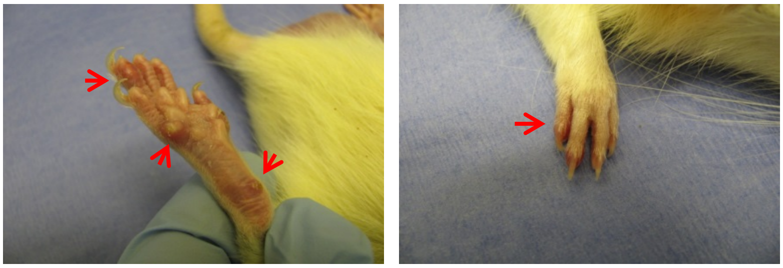

CpG

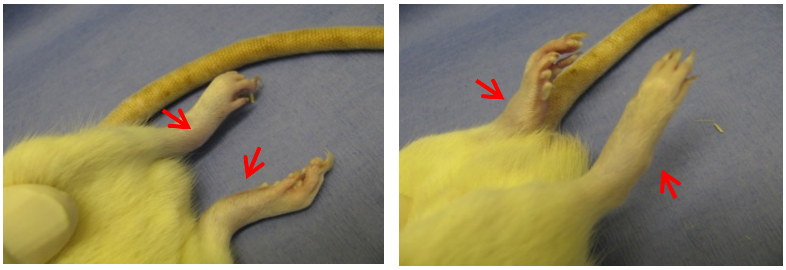

Suppl. Fig. 2 Immunization with flagellin and CpG induced arthritis in HLA-B27 tg rats. HLA-B27 tg rats were immunized with 50 µg flagellin in 100 µl IFA or 50 µg CpG in 100 µl IFA, arthritis symptoms, as indicated by the red arrows in the pictures, could be observed.
